# Early allogeneic stem cell transplantation after melphalan-induced aplasia leads to long-term survival in patients with primary refractory AML

**DOI:** 10.1007/s00277-025-06728-w

**Published:** 2025-11-14

**Authors:** Claudius Söhn, Kerstin Schäfer-Eckart, Knut Wendelin, Stefan Knop

**Affiliations:** Department of Internal Medicine, Hematology and Oncology, Paracelsus Medical University, Klinikum Nuremberg, Nuremberg, Germany

**Keywords:** Primary refractory, AML, Melphalan, Aplasia, Allogeneic hematopoietic stem cell transplantation

## Abstract

The prognosis of patients with AML, who fail to achieve complete remission after induction therapy (= primary refractory disease) has been dismal for many years. Allo-HSCT is the only curative approach and standard of care for eligible patients in this group. The need for inducing a remission before transplantation in primary refractory patients has not been finally clarified. The objective of this retrospective single-center evaluation is to analyze the outcomes of primary refractory patients who underwent immediate allogeneic HSCT after chemotherapy induced aplasia in the context of the conditioning regimen in a real-life setting. We started a program of immediate transplantation policy for eligible patients with refractory AML after induction chemotherapy in our center in 2010. All patients with a diagnosis of primary refractory AML who underwent their first allogeneic hematopoietic stem cell transplantation in active disease between 03/2010 and 07/2023 at the department of Hematology at Klinikum Nuremberg, Germany, were included and analyzed. 50 patients were included in this analysis. Mean time from start of induction treatment to transplantation was 3 months (range 1.4–7.8 months). Melphalan (100–140 mg/m^2^) for induction of aplasia was used in 45/50 pts (90%) prior to conditioning. After a median follow-up of 9.97 (range: 0.03–143.8) months 18/50 (36%) patients were alive without relapse. Median and mean relapse free survival were 9.97 (range: 0.03–143.8) and 28.13 (range: 0.03–143.8) months respectively. The median and mean OS were 9.97 (range: 0.03–143.8) and 28.64 (range: 0.03–143.8) months. The 100-day, 1-year and 2-year survival rates were 66%, 48% and 42% respectively. Cumulative incidence of relapse at 2-years after transplantation was 18.5% (95% C.I., 10.3% − 33.1%) with a median time from HSCT to relapse of 4.85 (Range: 0.67-18,3) months. Cumulative incidence of non-relapse mortality at 100 days, 1- year and 2-years was 26% (95% C.I., 16.3% − 41.5%), 36.3% (C.I., 25.1% − 52.5%) and 38.4% (95% C.I., 26.9% − 54.7%) respectively. Main cause of overall mortality was TRM (58.1%) with Sepsis (41.9%) being the biggest single contributor. The only significant independent factor associated with decreased overall and 100-day survival was a positive patient CMV-status (independent of donor CMV-serostatus). The observed survival-rates in our study compare favorably to those published for established salvage-therapy regimens as well as other retrospective studies analyzing HSCT in active disease. We demonstrate that allogeneic hematopoietic stem cell transplantation in active disease is a reasonable approach for patients with primary refractory AML offering the chance for long-term survival for about one third of patients. The effect is compromised by the high incidence of non-relapse mortality.

## Introduction

Acute myeloid leukemia (AML) is the most common form of leukemia in adult patients. Over the last decades survival rates of younger patients with newly diagnosed AML have improved mainly because of earlier timing of allogeneic hematopoietic stem cell transplantation (Allo-HSCT), better donor selection and advances in supportive care [1]. About 20–40% of younger patients (18–60 years) and about 40–60% of older patients (>60 years) however, fail to achieve complete remission (CR) after induction therapy (= primary refractory disease) and 50–70% of patients, who achieve complete remission will relapse [2]. The prognosis of those patients has been dismal for many years. Allo-HSCT is the only curative approach and standard of care for eligible patients in this group [3]. The need for inducing a remission before transplantation in primary refractory patients has not been finally clarified, but the results of the prospective, randomized trial recently published by Stelljes et al. support the concept of an “immediate transplantation” without remission-inducing chemotherapy before [4].

We started a program of immediate transplantation policy for eligible patients with refractory AML after induction chemotherapy in our center in 2010. The objective of this retrospective single-center evaluation is to analyze the outcomes of primary refractory patients who underwent immediate allogeneic HSCT after chemotherapy induced aplasia in the context of the conditioning regimen in most of these patients in a real-life setting. In addition, we try to identify factors influencing prognosis in this setting.

## Methods

### Data collection and study population

In this retrospective single center study, all patients with a diagnosis of primary refractory AML who underwent their first allogeneic hematopoietic stem cell transplantation in active disease between 03/2010 and 07/2023 at the department of Hematology at Klinikum Nuremberg, Germany, were included. Patients were defined as primary refractory by failure to reach a first complete remission (bone marrow blasts < 5%) after one or two cycles of induction therapy according to European leukemia network (ELN) guidelines.

Patient characteristics are shown in Table 1. ELN risk classification was defined according to the 2022 ELN Guideline [5]. All patients, who had been classified according to guidelines in place at their diagnosis, were retrospectively classified according to the now valid 2022 ELN guideline.

**Table 1:** Patient characteristics of the 50 patients included in the study. ELN risk category was defined according to the ELN 2022 guideline

Patient characteristics	
All patients	*n* = 50
Gender	
male	30 (60%)
female	20 (40%)
Median age (years, yrs)	55.7 (Range: 24–75)
AML type	
AML	34 (68%)
sAML	10 (20%)
tAML	6 (12%)
ELN Risk category	
adverse	30 (60%)
intermediate	17 (34%)
favourable	3 (6%)
Blast count before conditioning	
> 30%	26 (52%)
≤ 30%	24 (48%)
EBMT Risk-Score (mean)	4.8 points (Range: 3–6)

All necessary data were retrospectively collected in anonymized form from the hospital information system at Klinikum Nuremberg and the internal documentation system of the department. All patients provided written informed consent to analysis of their medical data at inclusion to the AML-registry of the Study alliance leukemia (SAL). The study was examined and approved by the local, internal review board.

Variables used in this study included patient and disease characteristics (gender, sex, date of diagnosis, patient age at HSCT, AML subtype, cytogenetic risk group at diagnosis, blast count before conditioning, EBMT risk-score), treatment before transplantation (Number of induction cycles, induction regimen) and transplantation characteristics (Time between diagnosis and HSCT, donor type, graft source, conditioning regimen, Graft-versus-host-disease (GvHD)-prophylaxis, Recipient/Donor-Blood-Type-Match, CMV-Match, number of CD34+-transplanted cells) and follow-up parameters (time to blood count regeneration, remission-status post-HSCT, acute and chronic GvHD, 100 day survival, 2-year survival, overall survival (OS), relapse-free survival (RFS), relapse incidence (RI), non-relapse mortality (NRM), Treatment-related mortality (TRM), refined Graft-versus-host disease-free, relapse-free survival (rGFRS), cause of death).

### Definitions

OS was defined as time between transplantation and death from any cause or censoring at last follow-up. RFS was defined as time to documented relapse or death. Refined GFRS was defined as time between transplantation and survival without grade III-IV acute GVHD, severe chronic GVHD, disease relapse or death from any cause.

Relapse incidence was defined as confirmation of leukemic blasts in the peripheral blood or bone marrow > 5% after previously achieving a complete remission. Grading of acute GvHD was documented according to the modified Glucksberg criteria into Grades I-IV. Grading of chronic GVHD into limited or extensive was based on the modified Seattle criteria.

Blood count regeneration was defined for neutrophils as the first of three consecutive days with an absolute neutrophil count (ANC) of at least 0.5 × 10^9^/L and for platelets as the first of 7 consecutive days with a platelet count of 20 × 10^6^/L or higher in the absence of platelet transfusion [6]. The last assessment of follow-up data was performed in 12/2024.

### Statistical analysis

Composite survival outcomes (OS, rGFRS and RFS) were evaluated using Kaplan-Meier-models. Binary Logistic regression analysis was performed to evaluate variables influencing 100 d survival. Univariate analysis for survival outcomes was done for categorical variables using the Breslow-estimator. For numerical variables linear regression analysis was applied. A Cox proportional hazard model for multivariate analysis of variables influencing time-to-event data was not performed because of the small sample size and uneven distribution in the different categories leading to unstable estimates of Hazard ratios and over-/or underestimation of effects.

Relapse incidence and NRM are reported as cumulative incidence estimates calculated using an appropriate competing risk model in accordance with the statistical methodology suggested for European Group for Blood and Marrow Transplantation (EBMT) studies published in 2013 by Simona Iacobelli on behalf of the EBMT Statistical Committee [6, 7]. The level of significance was set at *p* < 0.05. Statistical analysis was performed using SPSS Statistics (IBM Corp. Released 2021. IBM SPSS Statistics for Windows, Version 28.0. Armonk, NY: IBM Corp) and XLSTAT (Addinsoft. XLSTAT statistical and data analysis solution, Version 2024.3.0., Paris, France).

## Results

### Patient and disease characteristics

A total of 50 patients were included in this analysis. Median age at transplantation was 56 (range, 24–75 years) and 60% of the patients were male. 30 patients had adverse-, 17 patients intermediate- and 3 patients favorable- risk cytogenetics according to the 2022 ELN Classification [5]. 52% of patients had a bone marrow blast count of over 30% before start of conditioning therapy. The mean EBMT risk-score for transplantation was 4.8 points.

### Treatment before transplantation and transplantation characteristics

All treatment and transplantation characteristics are shown in Table 2. All patients received one cycle of Cytarabine and Daunorubicin (DA 3 + 7) as first induction therapy, some patients in combination with Gemtuzumab-Ozogamicin (GO) or Midostaurin because of CBF-AML (GO) or FLT-3 ITD/TKD (Midostaurin) mutations. 30 patients received a second cycle of chemotherapy as re-induction after failure to reach complete remission with first induction therapy. In 90% of these patients, high-dose Cytarabine (cumulative dose between 6 g/m^2^−18 g/m^2^) was administered. Two patients received a second application of DA (either as DA 7 + 3 or as fixed combination), 1 patient received Azacitidine.

**Table 2:** Treatment characteristics of the 50 patients included in the study

Treatment characteristics	
**All patients**	*n* = 50
**Induction Therapy**	
**Induction I**	*n* = 50
DA 3 + 7	42
DA 3 + 7 + Midostaurin	2
DA 3 + 7 + Mitoxantron	1
DA 3 + 7 + GO	1
Daunorubicin/cytarabine fixed combination + Mido	1
Daunorubicin/cytarabine fixed combination	3
**Induction II**	*n* = 30
HDAraC	24
DA 3 + 7	1
HDAraC + Clofarabine	1
HDAraC + Midostaurin	2
Azacitidin	1
Daunorubicin/cytarabine fixed combination	1
**Conditioning**	
MelTreoFlu	36 (72%)
MelTBIFlu	5 (10%)
MelBuFlu	3 (6%)
TBIFlu	2 (4%)
BuFlu	2 (4%)
TreoFlu	1 (2%)
FLAMSA-RIC	1 (2%)
**ATG Prophylaxis**	
yes	41 (82%)
no	9 (18%)
**Donor**	
MRD	8 (16%)
MUD	25 (50%)
MMUD	14 (28%)
Haploident	3 (6%)
**CMV-Status**	
P-/D-	14 (28%)
P-/D+	4 (8%)
P+/D-	16 (32%)
P+/D+	16 (32%)
**CD 34 + Cell Count of graft**	7.0*10^6 (Range: 3.06–10.01*10^6)

Melphalan (100–140 mg/m^2^) for induction of aplasia was used in 45/50 pts (90%) prior to conditioning. The conditioning regimen consisted of Treosulfan (30 g/m) and Fludarabine (150 mg/m) in most pts (74%).

25/50 (50%) patients had a matched unrelated donor (MUD), 14/50 (28%) had a mismatched unrelated donor (MMUD, 1 antigen or allele mismatch), 8 (16%) patients had a matched related donor (MRD), and 3 (6%) patients received a haploidentical transplantation from a related donor. Mean time from start of induction treatment to transplantation was 3 months (range 1.4–7.8 months). All patients received G-CSF stimulated peripheral blood stem cells. GvHD-prophylaxis consisted of Cyclosporin and Methotrexat or Cyclosporin and Mycophenolatmofetil. 41/50 (82%) patients additionally received ATG. No patient received prophylactic donor lymphozyte infusion (DLI) after transplantation.

The median time of neutrophil regeneration was 17 (range: 7–32) days, the median time of platelet regeneration was 21 (range: 13–89) days. 10 Patients did not reach regeneration of either neutrophils, platelets or both due to either early treatment-related death (*n* = 8; PERDS *n* = 1, Sepsis *n* = 6, graft failure *n* = 1) or early disease-related mortality (*n* = 2; refractory disease *n* = 2).

### Transplantation outcomes

#### aGvHD, cGvHD refined GFRS

Grade III-IV aGvHD was documented in 4 patients (8%), all of whom presented with skin involvement, with additional Grade III-IV GI-involvement in 3 patients. In 4 out of the 33 patients (12%), who achieved 100 d survival, extensive cGvHD was documented. All these 4 patients had received HSCT from a MUD. Median refined GFRS was 6 months (range: 0.03–143.8 months).

#### OS, RFS

After a median follow-up of 9.97 (range: 0.03–143.8) months 18/50 (36%) patients were alive without relapse. Median and mean relapse free survival were 9.97 (range: 0.03–143.8) and 28.13 (range: 0.03–143.8) months respectively. The median and mean OS were 9.97 (range: 0.03–143.8) and 28.64 (range: 0.03–143.8) months.

**Fig. 1:**
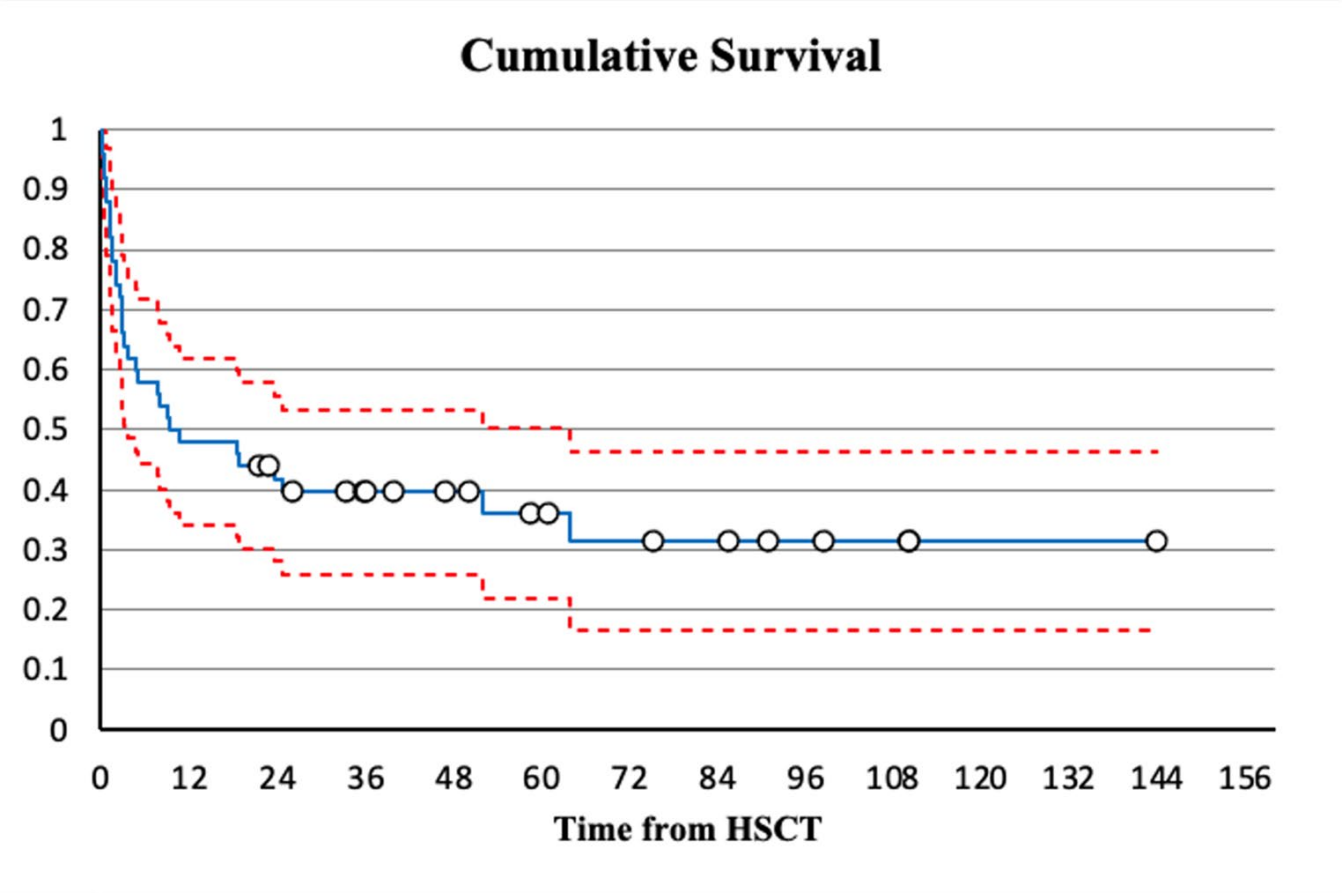
Kaplan-Meier plot of the cumulative survival in the whole cohort

The 100-day, 1-year and 2-year survival rates were 66%, 48% and 42% respectively. Figure 1 displays the Kaplan-Meier plot of the cumulative survival in the whole cohort.

There was no significant difference in OS, RFS and rGRFS respectively between patients receiving 1 induction vs. 2 induction cycles (*p* = 0.40; *p* = 0.52; *p* = 0.67), gender (*p* = 0.62; *p* = 0.80; *p* = 0.81), ATG application (*p* = 0.06; *p* = 0.15; 0.12), Donor type (*p* = 0.81; *p* = 0.79; *p* = 0.91), AML type (*p* = 0.24; *p* = 0.12; *p* = 0.21), bone marrow blast count before start of conditioning therapy (*p* = 0.41; *P* = 0.45; *P* = 0.66) or ELN category (*p* = 0.69; *p* = 0.72; *p* = 0.57).

**Fig. 2:**
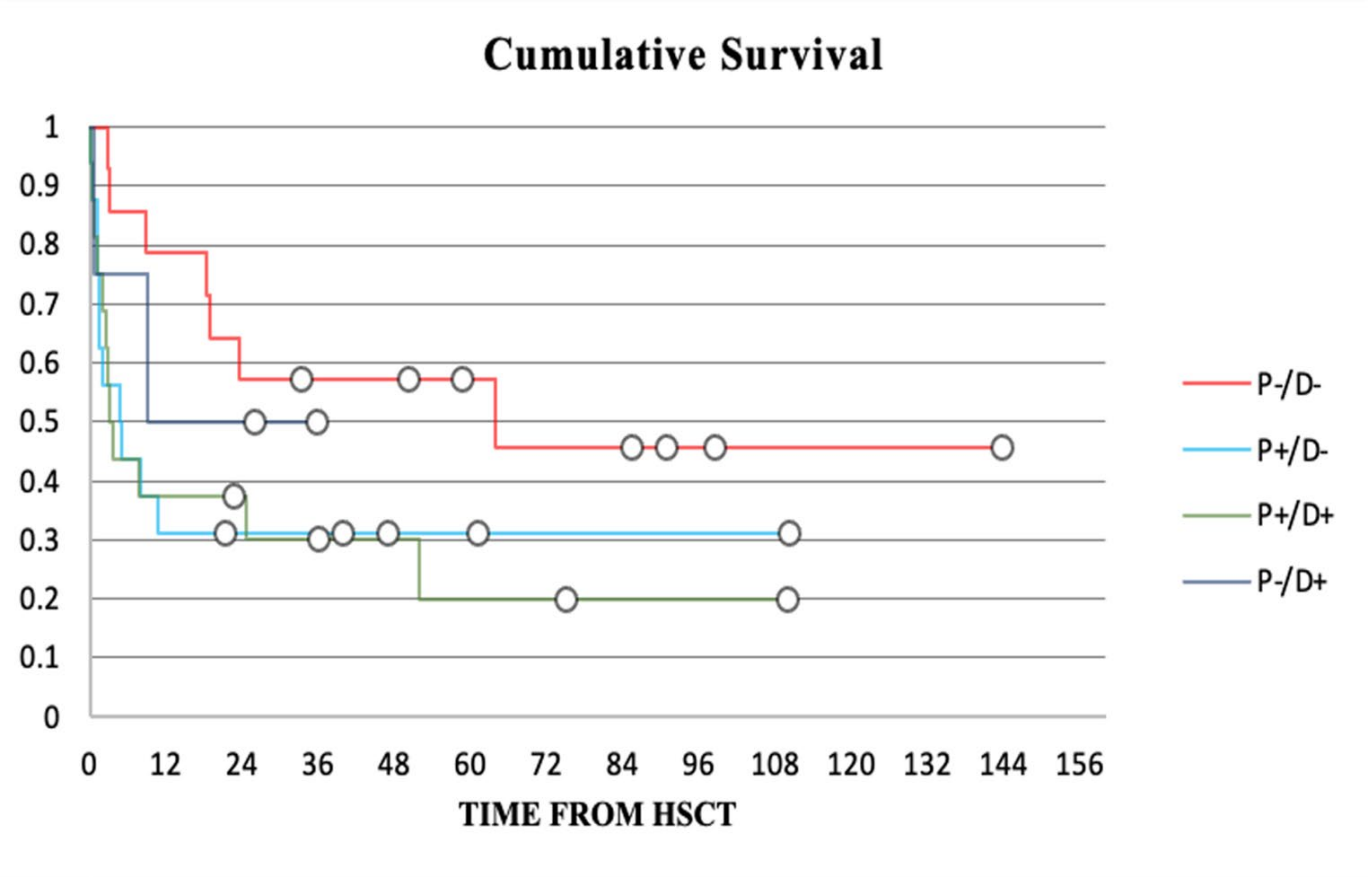
Kaplan-Meier plot of the cumulative survival depending on patient/donor-CMV-status

Pairwise comparison of the OS distribution using the Breslow estimator revealed a statistically significant difference in the survival distributions depending on CMV-status. P-/D- CMV match was associated with significant longer OS (Fig. 2) in comparison to P+/D- (X^2^ = 5.4; *p* = 0.02) and P+/D+ (X^2^ = 5.2; *p* = 0.02).

Pearson correlation for continuous variables showed no significant correlation between OS/RFS/rGRFS and time between diagnosis and HSCT (r: 0.037, *p* = 0.80; r: 0.033, *p* = 0.82; r: 0.103, *p* = 0.48), age at HSCT (r: 0.067, *p* = 0.64; r: 0.063, *p* = 0.67; r: 0.163, *p* = 0.26) or CD34 + cell count (r: 0.013, *p* = 0.93; r:0.018, *p* = 0.90; r: −0.023, *p* = 0.87).

Binary Logistic regression showed no significant impact of time from diagnosis to HSCT (*p* = 0.69), gender (*p* = 0.94), age at HSCT (*p* = 0.13), AML type (primary AML: *p* = 0.19; tAML: *p* = 0.07; sAML: *p* = 0.65), ELN risk category (adverse: *p* = 0.52; intermediate: *p* = 0.25; favorable: *p* = 0.99), Donor type (MUD: *p* = 0.84; MRD: *p* = 0.84; MMUD: *p* = 0.95; haploident: *p* = 0.28) or ATG application (*p* = 0.14) on 100 d survival. The only variables significantly associated with decreased chance of 100 d survival were P+/D- (Odds ratio 0.003, 95% C.I.: 0.00–0.57.00.57; *p* = 0.03) and P+/D+ (Odds ratio 0.003; 95% C.I.: 0.00–0.51.00.51; *p* = 0.03) CMV match.

#### Remission rates, relapse incidence and NRM

In 39/50 patients (78%), complete remission could be documented after HSCT. Of the 11 patients not achieving CR, 9 patients (18%) died of early transplant-related complications before CR could be evaluated. 2 patients (4%) had refractory disease.

**Fig. 3:**
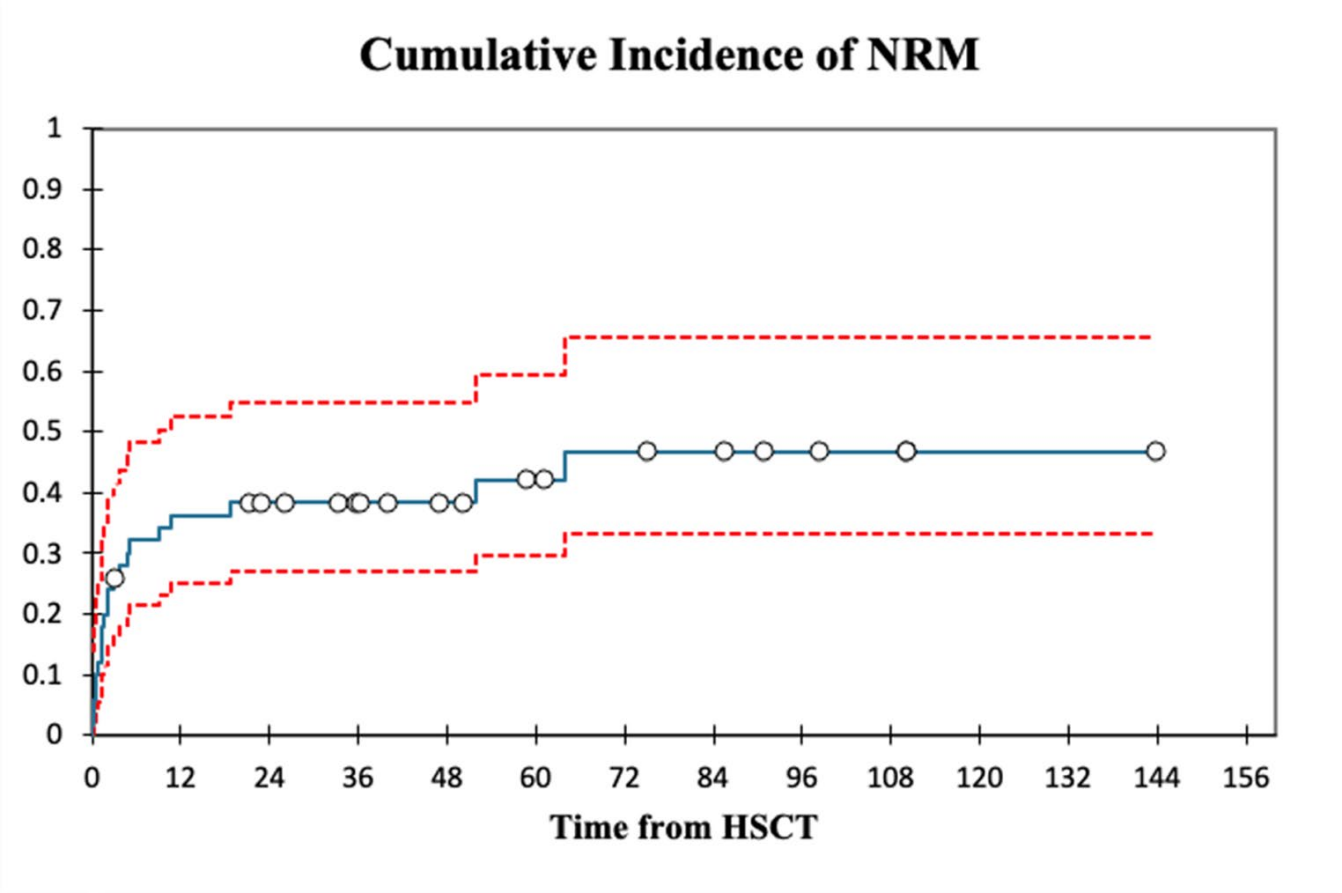
Cumulative incidence of non-relapse mortality

**Fig. 4:**
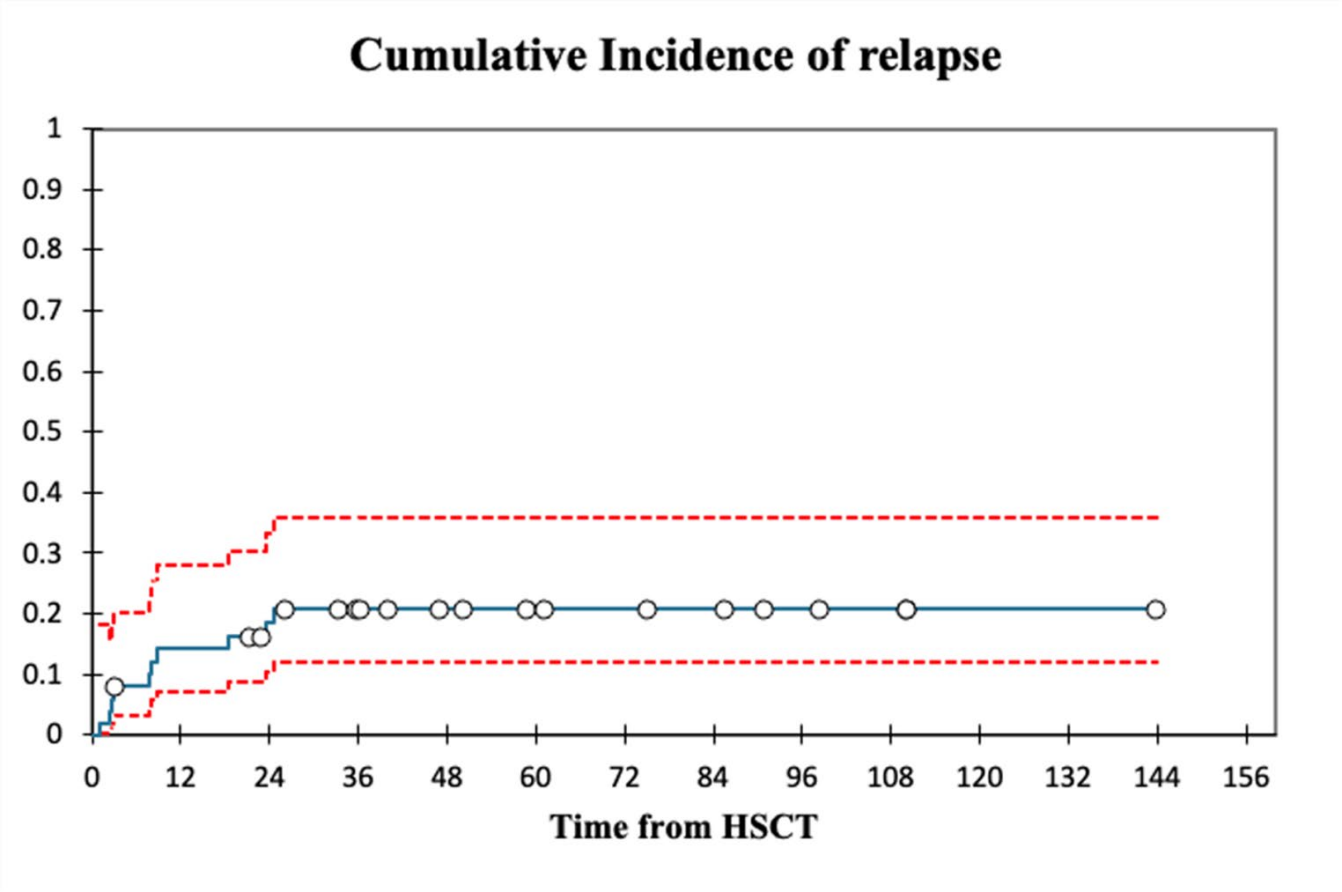
Cumulative incidence of relapse

Cumulative incidence of relapse at 2-years after transplantation was 18.5% (95% C.I., 10.3% − 33.1%) with a median time from HSCT to relapse of 4.85 (Range: 0.67–18.67,3) months. Cumulative incidence of non-relapse mortality at 100 days, 1- year and 2-years was 26% (95% C.I., 16.3% − 41.5%), 36.3% (C.I., 25.1% − 52.5%) and 38.4% (95% C.I., 26.9% − 54.7%) respectively. Figures 3 and 4 illustrate the cumulative incidences of non-relapse mortality and relapse. Main cause of overall mortality was TRM (58.1%) with Sepsis (41.9%) being the biggest single contributor. Two patients died of non-treatment/non-disease related causes (Colon-cancer over 5 years after transplantation and COVID-19 over 4 years after transplantation).

## Discussion

The goal of this retrospective single-center evaluation was to analyze the outcomes of patients with primary refractory AML after early allogeneic HSCT in active disease without a remission induction or salvage chemotherapy separated from preparation regimen in our center in a real-life setting. Firstly, we could show that transplantation is feasible as fast as < 6 weeks after initiation of induction therapy due to high donor availability for most patients in Germany. In this cohort with an otherwise dismal prognosis, HSCT resulted in a 100-day survival rate of 66% and an estimated 2-year survival-rate of 42% offering patients a realistic chance of long-term survival despite failure of their initial treatment. These observed survival-rates in our study compare favorably to those published for established salvage-therapy regimens as well as other retrospective studies analyzing HSCT in active disease: Middeke et al. demonstrated an overall survival of 43% at 2 years after clofarabine based salvage-therapy [3]. In a trial comparing Quizartinib versus salvage chemotherapy in relapsed or refractory FLT3-ITD AML Cortes et al. published a 2-year survival rate of 20% after salvage therapy of investigators’ choice. For HSCT in active disease using Treosulfan/Fludarabine based conditioning after high-dose Melphalan, Steckel et al. demonstrated 1-year OS rates of 46% [8]. An analysis from the Acute Leukemia Working Party of the European Society for Blood and Marrow Transplantation of FLAMSA-Based Reduced-Intensity Conditioning versus Myeloablative Conditioning in Younger Patients with Relapsed/Refractory Acute Myeloid Leukemia with active disease at the time of HSCT showed 2-year OS rates of 36% for FLAMSA-TBI-based conditioning and 50% for FLAMSA-chemotherapy based conditioning [9]. In a direct comparison of patients receiving a preconditioning intervention (PCI) vs. patients reaching no PCI Tachibana et al. showed comparable 2-year OS results for patients receiving no PCI (35.1%) and patients with a good response to PCI (34.4%). Patients with a poor response to PCI had significantly worse 2-year OS rates (14%) [10]. Similarly, Wattad et al. demonstrated that patients who did not achieve complete remission after intensive salvage therapy had significantly worse outcomes than patients transplanted without receiving salvage therapy before HSCT, strengthening the hypothesis that the immune effect of upfront HSCT is most likely the main driver for achieving disease remission. This approach can be regarded as the mainstay of treatment for this chemotherapy-refractory cohort [11]. These results are mirrored by the first prospective, randomized and recently published study by Stelljes et al., where allogeneic HSCT in an “as-soon-as-possible” approach was non-inferior to a remission induction strategy [4]. 

Inducing complete remission by intensive salvage chemotherapy is associated with considerable treatment related complications and only about 20–50% of patients will achieve CR after salvage chemotherapy [12, 13]. Besides increasing toxicity, increasing mutational burden after prolonged courses of chemotherapy as has been shown in a study from Krönke et al. may furthermore argue for an immediate transplantation [14].

Re-induction after failure to reach complete remission after first induction did not add a survival benefit in our cohort, supporting the notion that transplantation is the main driver of survival for patients refractory to their first line chemotherapy. Bone marrow blast count (>30% or ≤ 30%) before start of conditioning therapy had no significant effect on OS, RFS or rGRFS in our analysis. This is in accordance with a study by Sockel et al., where variables predicting outcome in this setting of sequential conditioning therapy, with a focus on pretreatment morphologic blast count were investigated. Here a significant influence could only be seen for patients with a pre-transplantation blast count >20% in comparison to patients with a blast count of < 5%, but not to patients with a blast count of 5–20% [15].

The results also showed that effectivity of our approach is compromised by the high rate of early transplant-related morbidity mainly because of infectious reasons. The main reason is the long period of profound neutropenia, mainly because of the refractory disease status. Whether the administration of melphalan 10–14 days prior conditioning further adds to the high infection rate cannot be answered, as most patients received this aplasia-inducing chemotherapy. On the other hand, the promising transplantation results for refractory patients published in the last years were all done with aplasia inducing regimens to enhance the antileukemic effect of the conditioning therapy.

The only significant independent factor associated with decreased overall and 100-day survival in our study was a positive patient CMV-status (independent of donor CMV-serostatus). This is in accordance with previous studies, where CMV-seropositivity has been described as an independent risk factor for decreased OS and higher NRM [16, 17]. No patient died of CMV-infection related causes.

Of note, retrospective analyses are prone to selection bias. In this study we analyzed all consecutive patients, who were transplanted in active disease at our center between 03/2010 and 07/2023. Yet, initial selection of patients suitable for transplantation is influenced by many factors, including patient’s preference and physician’s discretion introducing a potential selection bias. In addition, with such a small sample size all findings must be interpreted with caution since statistical power is limited. Especially in subgroup analysis a potential bias through over-/or underestimation of effects can be introduced. To our opinion, including patients over a span of 13 years, 50 patients still give a meaningful representation of this patient cohort from a single center. Furthermore, our results compare to those of retrospective as well as prospective studies, which included a bigger sample size, indicating reproducibility.

In this retrospective single-center analysis, we demonstrate that allogeneic hematopoietic stem cell transplantation in active disease is a reasonable approach for patients with primary refractory AML offering the chance for long-term survival for about one third of patients with an otherwise dismal prognosis and thus contradicts widespread therapeutic nihilism. Future research will need to address the need for optimized supportive care given the high incidence of non-relapse mortality.

## Data Availability

The data that support the findings of this study are not openly available due to reasons of sensitivity and are available from the corresponding author upon reasonable request.

## Abbreviations

AML: Acute Myeloid Leukemia
Allo-HSCT: Allogeneic Hematopoietic Stem Cell Transplantation
CR: Complete Remission
ELN: European Leukemia Network
GvHD: Graft versus Host Disease
OS: Overall Survival
RFS: Relapse Free Survival
RI: Relapse Incidence
NRM: Non-Relapse Mortality
TRM: Treatment Related Mortality
rGFRS: Refined Graft-versus-host disease-free, Relapse-free Survival
EBMT: European Group for Blood and Marrow Transplantation
CBF: Core Binding Factor
FLT3-ITD/TKD: FMS-related Tyrosine Kinase-3 Internal-tandem Duplication/Tyrosine-kinase Domain
MUD: Matched Unrelated Donor
MRD: Matched Related Donor
MMUD: Mismatched Unrelated Donor
ATG: Anti-thymocyte-globulin
PERDS: Peri-engraftment Respiratory Distress Syndrome
ANC: Absolute Neutrophil Count
HDAraC: High-Dose Cytarabine
TBI: Total Body Irradiation
BuFlu: Busulfan/Fludarabine
FLAMSA-RIC: Fludarabine, Cytarabine, and Amsacrine-Reduced-Intensity Conditioning
CMV: Cytomegalovirus
